# Molecular Stress Biomarkers in Aquaculture: A Systematic Review

**DOI:** 10.3390/biology15191681

**Published:** 2026-09-22

**Authors:** Alba Sellés-Egea, David G. Valcarce, Vanesa Robles

**Affiliations:** Cell Biology Area, Molecular Biology Department, Universidad de León, Campus de Vegazana s/n, 24071 León, Spain; asele@unileon.es (A.S.-E.); dgarv@unileon.es (D.G.V.)

**Keywords:** aquaculture, molecular biomarkers, fish welfare, production-derived stressors, teleost fish

## Abstract

This systematic review of the literature aimed to explore the molecular biomarkers used for the detection of stress in the aquaculture sector over the last decade. Relevant studies were retrieved from the Web of Science, Scopus and PubMed databases, which were last searched on 13 June 2025. After screening according to predefined eligibility criteria and PRISMA 2020, 64 peer-reviewed original research articles published between 2015 and 2025 were included. The included studies were qualitatively synthesised. These studies evaluated molecular biomarkers of stress across 31 fish species, with *Salmo salar* being the most frequently studied, followed by *Oncorhynchus mykiss*. China, Spain and Canada contributed the largest numbers of included studies. In total, 20 different stressors were identified. Temperature was the most frequently investigated stressor, followed by stocking density, hypoxia and exposure to various chemicals. Publication activity has grown over the last decade, with a clear increase since 2020. This systematic review was conducted to integrate current knowledge and emerging evidence on molecular markers in teleost fish, aiming to provide a comprehensive understanding of both established biomarkers and novel molecular candidates identified through recent studies. Although considerable heterogeneity was observed among fish species, stress protocols and biomarker assessment methods, the evidence supports the growing value of integrated biomarker approaches for stress assessment in aquaculture. Overall, the evidence supports integrated, context-specific biomarker approaches that combine molecular endpoints with established physiological indicators to improve stress assessment in aquaculture.

## Simple Summary

Aquaculture is an important source of food worldwide, but fish are often exposed to stressful conditions such as transport, high stocking densities, handling and changes in water temperature. These situations can affect their health, welfare and growth. This systematic review examined research published during the last ten years to identify the main molecular biomarkers used to detect stress in fish. A total of 64 scientific studies covering 31 fish species were analysed. The results showed that commonly used indicators, such as stress hormones and proteins involved in cellular protection, remain valuable tools for assessing stress. At the same time, newer indicators based on non-coding RNAs (ncRNAs), particularly miRNAs, are emerging as promising alternatives because of their regulatory roles in gene expression. The review also found that combining several biological indicators provides a more consistent evaluation of fish welfare than relying on a single measure. This review highlights the sustained efforts to develop and optimise molecular stress markers for aquaculture applications, supporting improved stress monitoring and management, enhancing fish welfare, and contributing to more sustainable fish production for a growing global population.

## 1. Introduction

Aquaculture is currently experiencing rapid growth and has become an economic sector of great importance, not only as a source of employment and economic development, but also as an essential provider of protein for human nutrition [1]. It plays a major role in food security and, consequently, in the nutrition of millions of people around the world [1]. A major concern for the aquaculture industry, consumers and regulatory bodies is the physiological stress experienced by animals, since it directly influences growth performance, health status, product quality, overall production levels and animal welfare [1].

Within this industry, a wide variety of production-derived stressors have been identified that challenge animal homeostasis, such as transport (e.g., the transport of fingerlings to a distant culture system [2]), high stocking density (a key factor in maximising production and profitability, as farmers aim to optimise biomass per water volume [3]), handling (an unavoidable stressor given current facilities and processes [4]), food deprivation [5] or the introduction of pathogens in the system [6]. In addition, specimens may be exposed to a wide variety of environmental stressors, including temperature changes [7], chemical pollutants [8] and fluctuations in oxygen concentration [9]. Understanding the biological mechanisms underlying the stress response is therefore essential as it provides the basis for developing strategies that mitigate negative consequences and improve production efficiency and sustainability. Consequently, there has been a marked increase in the number of studies focusing on stress in fish, with the aim of identifying reliable indicators and management practices that improve welfare and productivity in aquaculture systems.

Stress is defined as the organismal response to a stimulus that is recognised as a threat, and represents an adaptive response to danger [10]. As a result, exposure to different stressors causes biochemical and physiological changes in organisms [8,11]. It acts through various pathways that affect the central nervous system (CNS), altering the expression of key genes such as corticotropin-releasing hormone (*crh*) [12], glucocorticoid receptor (*nr3c1*) or melanocortin 2 receptor (*mc2r*) [13], and causing changes in the production of hormones and neurotransmitters [14]. The primary stress response in fish involves rapid catecholaminergic signalling together with activation of the hypothalamic-pituitary-interrenal (HPI) axis, culminating in cortisol release [12].

The stress response is closely connected to the reproductive system through interactions between the HPI and brain–pituitary–gonadal (BPG) axes, linking stress physiology with reproductive regulation [15]. Consequently, prolonged stress can result in immunosuppression, reduced growth and reproductive dysfunction. In many teleost species, reproductive development is influenced by both genetic and environmental factors, meaning that stress may interfere with gonadal development and sexual differentiation, ultimately affecting reproductive performance and aquaculture productivity [15,16].

Stress can be categorised as acute or chronic [10]. Acute stress causes rapid changes in neuronal activity, resulting in a swift release of molecules and neurotransmitters with the aim of restoring organismal homeostasis as rapidly as possible. Conversely, chronic stress can produce sustained endocrine and transcriptional changes, including altered cortisol and leptin signalling and modulation of stress-related neuropeptide pathways such as Pomc and Npy [17,18]. Because of their multifactorial nature, stress markers can be assessed using metabolic, endocrine, behavioural, immunological and molecular indicators. Advances in molecular biology techniques have made gene expression analysis a valuable tool for evaluating physiological responses in animals, particularly in fish [19].

Conventionally, stress assessment in fish has relied on plasma cortisol and glucose, as well as haematological and hydro-mineral measurements. However, interpreting the results is challenging because stress responses depend on both intrinsic and extrinsic factors that interact through positive and negative feedback within hormonal pathways [4]. Extensive research has examined hormonal and metabolic homeostasis in plasma and whole-body responses to stressors. Understanding these regulatory factors and their transcriptional control provides valuable insights into the pathophysiology of stress [4]. Although these conventional indicators remain essential for assessing stress in fish, the present review focuses specifically on molecular biomarkers and considers physiological indicators only when they were evaluated alongside molecular endpoints.

The application of molecular biomarkers in aquaculture has attracted considerable interest, but their actual contribution to stress assessment remains under debate. During the last decade, the scientific community has investigated a wide range of biomarkers, including microRNAs, members of the heat shock protein family and stress-related key genes, frequently in combination with established physiological indicators such as cortisol. Although molecular markers are often presented as promising complements to conventional physiological or behavioural indicators, the evidence is still fragmented and, in many cases, restricted to experimental settings with limited transferability to farming practice. Moreover, variability in methodologies and the absence of standardised protocols have hindered comparisons across studies and slowed their integration into routine monitoring. In this context, a systematic evaluation of the literature is therefore required to clarify their relevance, identify gaps and provide guidance for their effective application in aquaculture. Accordingly, this review critically evaluates the use of molecular biomarkers for stress assessment in aquaculture-relevant teleost species or model species widely used in aquaculture research, while recognising the complementary role of well-established physiological indicators, such as cortisol, in their interpretation.

## 2. Materials and Methods

### 2.1. Search Strategy

The systematic review was reported in accordance with the Preferred Reporting Items for Systematic Reviews and Meta-Analyses (PRISMA) 2020 statement. The study selection process is summarised in the PRISMA flow diagram (Figure 1). A more detailed description of the methods (including the databases used, search string, inclusion and exclusion criteria and other details about study selection and data extraction and synthesis) is provided in Supplementary Table S1.

This systematic review was specifically aimed at identifying and evaluating studies investigating molecular biomarkers associated with stress responses in teleost fish relevant to aquaculture. For this purpose, we aimed to ascertain the maximum number of original articles published in peer-reviewed academic journals. Eligible studies were peer-reviewed original research articles published in English between 2015 and 13 June 2025. Studies had to investigate aquaculture-relevant teleosts or established experimental model species, apply an aquaculture-relevant stress protocol, and evaluate molecular biomarkers as a primary outcome. Studies assessing only physiological, biochemical or behavioural indicators were excluded (Table 1).

Eligible species included both commercially important aquaculture teleosts and widely used experimental model species, provided that their findings were considered relevant to improving the understanding or application of stress biomarkers in aquaculture.

Eligible stressors comprised conditions commonly encountered in aquaculture production or experimental settings, including handling, transport, stocking density, temperature fluctuations, hypoxia and other routine husbandry-related challenges. Studies addressing parasite infections as the sole experimental stressor were excluded, whereas infections evaluated together with other aquaculture-relevant stressors were considered eligible.

All studies included in this systematic review were primary experimental investigations conducted under controlled or semi-controlled experimental conditions, each applying specific stress protocols in teleost species. Review articles and purely conceptual papers were excluded in order to focus the analysis on current experimental evidence. This selection strategy provides a robust basis for interpreting biomarker responses over the last decade and for examining how their use has evolved.

Searches were performed in the electronic databases Web of Science, PubMed and Scopus, from 2015 to 13 June 2025. The core search strategy was: (“stress response” OR “physiological stress”) AND (“molecular markers” OR “biomarkers” OR “stress biomarkers”) AND (“fish” OR “teleost”) AND (“aquaculture” OR “fish farming”) AND (“gene expression” OR “transcriptomics”). Due to the specific characteristics of each search engine, the search string was adapted to each database. For Web of Science and Scopus, the search string was as follows: “TS = (“stress response” OR “physiological stress”) AND TS = (“molecular markers” OR biomarkers OR “stress biomarkers”) AND TS = (fish OR teleost) AND TS = (aquaculture OR “fish farming”) AND TS = (“gene expression” OR transcriptomics)”. The complete search strategy adapted for each database is provided in Supplementary Table S1 to facilitate reproducibility. Searches were restricted to peer-reviewed articles written in English; books, book chapters and conference proceedings were excluded.

Parsifal was used to manage the review protocol, database records, duplicate removal, screening and data extraction. After duplicate removal, three reviewers independently screened titles and abstracts against the predefined eligibility criteria. Potentially relevant articles underwent independent full-text assessment by the same three reviewers, and disagreements were resolved by consensus.

At the full-text eligibility stage, the methodological quality and applicability of potentially eligible studies were independently assessed by the same three reviewers using a predefined 12-item assessment implemented in Parsifal. Each criterion was scored as “Yes” (1 point), “Partially” (0.5 points), or “No” (0 points), resulting in a maximum possible score of 12. A predefined cut-off score of 10.5 was established for inclusion in the review; studies scoring below this threshold were excluded. The assessment covered methodological and applicability aspects including the evaluation of molecular stress biomarkers, adequacy and quantification of the stress protocol, relevance of the species and experimental conditions to aquaculture, presence of appropriate control groups, and complementary physiological or health-related variables. Disagreements between reviewers were resolved by consensus. The complete assessment criteria and scoring system are provided in Supplementary Table S2. Potential reporting bias, including selective outcome reporting and publication bias, was considered qualitatively; however, no formal assessment was performed due to the heterogeneity of the dataset and the absence of quantitative synthesis.

The review protocol was not prospectively registered. Nevertheless, the search strategy, eligibility criteria, study selection process and data extraction procedures were predefined before the literature search and applied consistently throughout the review.

### 2.2. Data Extraction

Once all the articles of interest had been compiled, data were extracted using a predefined data extraction form. The extracted information included the type of stressor analysed, the molecular biomarker assessed, the observed biological effects, the species studied and the applicability of the findings to the aquaculture industry. General bibliographic information about each article was also documented in an Excel spreadsheet including the article title, authors, year, journal, country of origin and study type. This workflow facilitated comparison and standardisation across studies, and enabled a transparent synthesis of the results. The study-level characteristics extracted from the 64 included articles, including species, stressor, biomarkers evaluated, acute/chronic classification, biological matrix or study material, and DOI, are provided in Supplementary Table S3. In this review, the country of origin for each article was assigned according to the affiliations of the first and last authors, as these usually reflect the primary institutional origins of the work. When clarification was needed, the corresponding author’s affiliation was also consulted and used to assign the study to a single country.

Because of substantial heterogeneity in species, stress protocols, biological matrices, biomarkers and analytical methods, a quantitative meta-analysis was not considered appropriate; findings were synthesised narratively.

For the purpose of synthesis, studies were grouped by tabulating the main extracted characteristics, including species, type of stressor, experimental design and molecular biomarkers analysed. These variables were compared against the predefined synthesis structure established prior to analysis. Studies were then assigned to synthesis groups based on similarity in biological model, stress condition and type of molecular response. When studies presented overlapping characteristics or did not clearly fit within a single category, classification decisions were reached by consensus among the three reviewers. This approach ensured a consistent and reproducible framework for data synthesis.

## 3. Results

A total of 64 studies were included in this systematic review, all meeting the established selection criteria. Of the 94 reports assessed at full text, 30 scored below the predefined methodological quality and applicability threshold of 10.5/12 and were excluded, resulting in 64 studies included in the qualitative synthesis (Figure 1). These articles covered the period from 2015 to 13 June 2025 and represented research conducted in 22 countries and involving 31 teleost species. Together, they provide a broad overview of the molecular biomarkers currently investigated for stress assessment in aquaculture. An overview of the stressors investigated, the selected stress indicators, their combinations, and the biological matrices used for cortisol measurement is provided in Figure 2. The temporal, taxonomic, geographical, and aquatic-environment distribution of the included studies is summarized in Figure 3. Publication activity increased overall during the study period, particularly from 2020 onwards, although substantial year-to-year variation was observed (Figure 3A). Geographically, China (14 studies), Spain (6 studies), Canada (5 studies), India (4 studies) and Chile (4 studies) were the main contributors to the available literature (Figure 3C). All included studies were experimental and were conducted under controlled or semi-controlled conditions, providing direct experimental evidence of stress-biomarker responses in teleost fish.

Thus, during this period, cortisol remained a frequently investigated endocrine stress indicator, whereas HSPs were among the most extensively studied molecular biomarkers. In contrast, microRNAs (miRNAs) have emerged more recently in the literature, specifically since 2021, and are rapidly gaining prominence as promising molecular indicators in this field. While cortisol and HSPs maintain a steady presence in research, the growing interest in miRNAs highlights their increasing potential for stress assessment (Figure 2B). Together, these findings illustrate the progressive diversification of biomarker research in aquaculture over the last decade.

Temperature emerged as the predominant stressor, being investigated in 31 of the 64 studies, which represents nearly half of the available evidence (Figure 2A). It was not only investigated as an isolated factor but was also frequently combined with other stressors, such as stocking density, hypoxia, handling, chemical exposure and salinity changes (Figure 2C).

A total of 31 species were identified across the included studies. Among them, *Salmo salar*, *Oncorhynchus mykiss*, *Cyprinus carpio* and *Dicentrarchus labrax* (Figure 3B) emerged as the most frequently investigated species, accounting for a substantial proportion of the available evidence. Of the 31 species represented in the articles included in this SLR, 16 were freshwater species, 11 were marine species and 4 were associated with both freshwater and marine environments (Figure 3D). This distribution shows a slight predominance of studies on freshwater species, while still encompassing key species of marine aquaculture. In general, although 31 teleost species were represented, the available evidence was concentrated in a relatively small number of species, with the remaining species being investigated in comparatively few studies (Figure 3B).

Overall, the studies included in this review reveal three major trends: (i) an overall increase in research activity, with marked year-to-year variation over the last decade; (ii) the predominance of temperature as the principal experimental stressor and (iii) the continued prominence of cortisol and heat shock proteins as the most widely investigated biomarkers, alongside the recent emergence of miRNAs as molecular indicators. In addition, the use of combined-stressor models reflects efforts to reproduce more complex experimental scenarios. Collectively, these findings summarise the main characteristics of the current evidence on molecular stress biomarkers in teleost aquaculture and provide the basis for the critical discussion presented in the following section.

## 4. Discussion

### 4.1. Main Stress-Assessment Methods

It is well known that stress is multifactorial in nature and, therefore, various approaches must be considered when studying the body’s response [20]. Although numerous physiological, endocrine, metabolic and behavioural indicators have been described for stress assessment in fish, the present review specifically focuses on molecular biomarkers and on studies in which molecular endpoints constituted the primary objective, while also considering established physiological indicators, such as cortisol, when evaluated alongside molecular responses. Accordingly, the following discussion focuses on the molecular biomarker categories most extensively represented in the selected literature, particularly heat shock proteins (HSPs) and miRNAs, while cortisol is considered separately as an established endocrine indicator frequently assessed alongside these molecular responses.

Among the established physiological indicators of stress, catecholamines and cortisol reflect key components of the primary endocrine stress response. However, because the present review was specifically focused on studies evaluating molecular endpoints, these conventional indicators were considered only when assessed alongside molecular biomarkers. Within the selected literature, cortisol was by far the most frequently represented of these physiological indicators and is therefore discussed here in greater detail. Cortisol is synthesised and released following activation of the hypothalamic–pituitary–interrenal (HPI) axis [10] and remains one of the most widely used physiological indicators of stress in aquaculture welfare assessments (Figure 2B). Among the studies retrieved, several evaluated cortisol; however, these studies frequently combined it with candidate molecular biomarkers to obtain a more comprehensive assessment of the stress response [21,22,23].

Within studies included in this review, plasma was the most commonly analysed matrix for cortisol quantification (Figure 2D), typically by immunoassay on samples obtained shortly after an acute challenge. However, plasma cortisol measurements may provide an incomplete picture of chronic stress, as they reflect only the endocrine status at the time of sampling and lack retrospective resolution. As an example, in *Salmo salar* (Atlantic salmon) exposed to prolonged stress, cortisol levels may decrease despite impaired growth and cardiac remodelling [24], indicating a dissociation between circulating cortisol and physiological condition.

Beyond plasma, additional non-lethal matrices have been explored in recent years. In Bortoletti et al. (2021) [25], a transport challenge in *Argyrosomus regius* (meagre) was associated with coordinated changes in muscle cortisol levels, glucocorticoid receptor expression and oxidative stress markers, indicating that muscle tissue reflects physiologically relevant aspects of the stress response. Other studies have examined cortisol in skin scales [24,26], which can accumulate circulating cortisol over time. Aerts et al. (2015) [26] demonstrated that cortisol measured in fish scales reflects systemic cortisol exposure over extended periods, providing a non-lethal matrix suitable for assessing chronic stress [26]. Moreover, evidence from Aerts et al. (2015) [26] and the subsequent study by Opinion et al. (2023) [24] showed that differences in scale cortisol may appear later than changes in plasma cortisol, supporting a temporal lag in scale incorporation during chronic stress. Consequently, while scale cortisol has limited resolution for acute stress responses, it provides a robust indicator of cumulative cortisol exposure and chronic stress history and it has considerable potential for linking chronic stress to long-term physiological changes [24]. Overall, cortisol remains one of the most widely used physiological indicators of stress in aquaculture welfare assessment, but its interpretation depends critically on the biological matrix and temporal sampling framework.

Whereas cortisol primarily reflects activation of the endocrine stress response, molecular biomarkers such as heat shock proteins (HSPs) provide complementary information on the cellular mechanisms activated in response to adverse conditions. HSPs are a family of molecular chaperones involved in a wide range of biological processes, including protein folding, assembly and cellular protection under adverse conditions [27]. Specifically, Hsp70 expression is frequently induced by thermal, environmental and pathological stressors and therefore is widely used as a biomarker of acute stress and physiological disturbances [27]. However, not all Hsp70 isoforms respond in the same way, as their sensitivity depends on the evolutionary group, the tissue, and the type of stress.

For example, in rainbow trout (*Oncorhynchus mykiss*) (a species particularly sensitive to temperature, food deprivation, and pollution), Hsp70a was the most responsive isoform across the stress conditions evaluated [27]. In the freshwater species *Perca flavescens*, Hsp70 levels increased in response to thermal stress at 26 °C, supporting its potential as an early biomarker of heat stress [4]. However, in species such as the European sea bass (*Dicentrarchus labrax*), no significant changes in Hsp70 were detected after a heat-stress protocol [28], whereas *hsp90aa1* was preferentially expressed at low temperatures (16 °C), suggesting a protective role against cold stress in this species [28].

Although HSPs are among the most extensively studied molecular biomarkers, their responses are strongly influenced by species, tissue, stressor type and exposure duration, limiting their value as universal indicators. This variability has driven the search for additional regulatory biomarkers, particularly microRNAs (miRNAs). MiRNAs have therefore emerged as promising molecular biomarkers for stress assessment in teleost fish, with an increasing number of studies published since 2021. Their growing interest is largely explained by their ability to regulate a wide range of biological processes, including development, sexual differentiation, immune responses and stress adaptation [29]. Accordingly, miRNAs provide valuable insights into the molecular mechanisms underlying stress responses and may complement conventional biomarkers by revealing early regulatory changes. An additional advantage of miRNAs is that some can be detected in extracellular fluids [30,31], where they are relatively stable and more resistant to degradation than conventional RNA molecules [32]. This characteristic makes them particularly promising as potential biomarkers for stress assessment in aquaculture. Unlike conventional physiological indicators, which mainly reflect physiological alterations after stress exposure, miRNAs provide information on the upstream molecular regulatory mechanisms underlying the stress response and therefore complement established biomarkers such as cortisol and HSPs.

However, despite their considerable potential, several challenges still limit the routine application of miRNAs in aquaculture. Although plasma-derived miRNAs provide valuable information on systemic physiological responses, the volume of blood required for miRNA analysis may limit non-lethal sampling in small fish species [33]. Skin mucus has therefore emerged as an attractive alternative because it can be collected non-lethally from smaller fish, although the biological significance of mucus-associated miRNAs still requires further investigation [33]. Emerging evidence suggests that mucus may contribute to the presence of extracellular miRNAs detected in the surrounding water, supporting the potential use of waterborne miRNAs as indicators of fish physiological status [33]. However, the exact origin and release mechanisms of these environmental miRNAs remain unclear, as mucus may represent only one of several possible sources, including other extracellular secretions or cellular material. Differences between miRNA profiles detected in plasma, mucus and water further suggest that the secretion and transport dynamics of miRNAs after stress exposure are not yet fully understood [33]. Therefore, comparative studies across different biological matrices will be essential to elucidate miRNA release pathways and determine the reliability of environmental miRNAs as stress biomarkers.

Beyond matrix selection, tissue- and species-specific expression patterns, together with the lack of standardised analytical methodologies and limited validation under commercial farming conditions, currently restrict their widespread implementation. Overall, current evidence suggests that miRNAs should not be considered replacements for conventional biomarkers but rather complementary components of multimarker approaches. Further cross-species validation and methodological standardisation will be essential before their routine incorporation into aquaculture welfare monitoring programmes.

Some of these complementary indicators are analysed in the context of each specific stress factor, where they were assessed alongside molecular biomarkers in the studies included in this review. Collectively, the evidence reviewed indicates that no single molecular biomarker provides a comprehensive assessment of stress across all biological contexts. Rather, molecular biomarkers such as HSPs and miRNAs, together with established endocrine indicators such as cortisol, should be viewed as complementary components that capture distinct levels of the stress response, from endocrine activation to cellular protection and post-transcriptional regulation. Their combined interpretation therefore offers a more comprehensive framework for stress assessment in aquaculture and provides the basis for evaluating their performance under different stressors, as discussed in the following sections.

### 4.2. Stressors

Under aquaculture conditions, handling and transport are two unavoidable stressors and rarely occur in isolation. Routine operations may involve capture, sorting, overcrowding, exposure to air, vibrations, water movement and changes in water quality [2,23,25]. Although temperature was the most frequently investigated individual stressor in the studies reviewed (Figure 2A), it was often evaluated together with hypoxia, stocking density or handling procedures (Figure 2C). This pattern reflects the multifactorial nature of stress in commercial aquaculture, where several challenges may act simultaneously or sequentially.

Among husbandry-related challenges, handling and transport are particularly relevant because fish may be exposed to a sequence of stressors before, during and after transfer. The pre-transport phase, which may include capture, crowding and handling, can make a substantial contribution to the overall stress response in the fish [23,25].

#### 4.2.1. Handling and Transport

Among studies addressing handling and transport, heat shock proteins (HSPs) have received particular attention [23,25,34,35,36]. Hsp70 and Hsp90 are the members most frequently evaluated in this context [34]. For example, *hsp70* is upregulated following the application of handling protocols, such as dip-net handling in *Dicentrarchus labrax* (European seabass) [35], whereas no significant Hsp70 response was detected in *Argyrosomus regius* (meagre) after an acute handling-related stress challenge [34].

Hsp90-related responses have also been reported following handling challenges. In *Solea senegalensis* (Senegalese sole), reduced human interaction resulted in downregulation of *hsp90aa* together with increased specific growth rate, body weight and body length [36]. These findings suggest that welfare-oriented refinements in routine protocols may translate into measurable improvements in productivity and HSP expression may be considered as a useful tool for the development of new culture protocols. However, the currently available evidence indicates that HSP responses depend strongly on the species and the characteristics of the handling protocol.

Beyond the cellular stress response reflected by HSPs, transport and handling-related stress also disrupts metabolism and ionic homeostasis. At the metabolic level, studies have identified changes in secondary metabolic responses, including an increase in glucose levels following acute stress [37]. In *Labeo rohita* (rohu), Biswal et al. (2021) examined glutamate pyruvate transaminase (Gpt), lactate dehydrogenase (Ldh), and malate dehydrogenase (Mdh) as secondary stress markers, suggesting the activation of gluconeogenesis and lipid mobilisation [2]. NaCl supplementation (0.4%) attenuated disturbances in ionic balance and improved several serum and tissue indicators relative to untreated transported fish [2].

These metabolic disturbances are accompanied by changes in oxidative stress pathways, indicating that the response to handling and transport extends beyond endocrine and energetic adjustments. Increased production of reactive oxygen species may overwhelm cellular antioxidant defences, leading to lipid peroxidation, protein damage and DNA injury [23]. Accordingly, markers such as Gsh-px (Glutathione peroxidase) [23], Cat (Catalase) [23,34], T-AOC (Total antioxidant capacity) [23], MDA (Malondialdehyde) [23], NO (Nitric oxide) [23] and 8-OHdG (8-hydroxy-2′-deoxyguanosine) [25] have been evaluated as indicators of transport-related stress. For example, transported fish showed increased antioxidant enzyme activity together with higher MDA and NO levels, suggesting activation of the antioxidant defence system in response to stress in some studies [23], whereas nitrotyrosine (NT) immunostaining revealed localised oxidative damage in the skin and gills in others [25]. By contrast, HNE and 8-OHdG did not consistently differ between stressed and control fish [25]. These results indicate that oxidative responses are an important but variable component of handling and transport stress, depending on the species, tissue, sampling time and experimental protocol.

Because biomarker responses evolve over time, the post-transport recovery period is essential for distinguishing transient adaptive responses from persistent physiological disruption. Several biomarkers returned to basal values within hours or days, which suggests that the effects of transport can be reversible [23]. Even so, not all parameters recover at the same rate; cortisol and metabolic markers may return to baseline earlier than markers related to oxidative stress or cellular responses [23].

By sampling fish at multiple time points following transport, including early (hours) and later (days) stages, Refaey and Li (2018) demonstrated that different biomarkers follow distinct recovery dynamics [23]. Although transport-derived stress may not cause lasting damage in every case, it can still leave fish in a vulnerable state for a period [23], with potential implications for subsequent resilience and susceptibility to additional stressors.

Taken together, handling and transport elicit coordinated endocrine, metabolic, oxidative and cellular responses whose magnitude and duration vary among species and experimental protocols. Circulating cortisol and glucose can capture rapid responses, whereas HSPs, oxidative markers, tissue cortisol and gene-expression profiles provide complementary information on cellular damage, adaptation and recovery. Their temporal dynamics also indicate that single-time-point assessments may underestimate the full impact of transport. Consequently, multimarker approaches incorporating appropriate recovery time points are likely to provide a more reliable basis for welfare monitoring than any individual endpoint.

#### 4.2.2. Stocking Density

Whereas handling and transport are generally episodic challenges, stocking density acts continuously throughout rearing and can modify stress physiology through crowding, resource competition and social interactions. Its effects are therefore strongly dependent on species, developmental stage and husbandry conditions [22].

In *Dicentrarchus labrax* exposed to different stocking densities, circulating miR-143-3p, miR-155-5p, miR-200a-3p, miR-223-3p and miR-205-1-5p have been reported to vary in association with stress and cortisol dynamics [21]. Functional analysis suggests that these miRNAs are involved in processes associated with the stress response, such as energy balance, angiogenesis and blood regulation, supporting their potential as complementary indicators of density-related stress.

In *Salmo salar*, classic markers such as cortisol exhibited density-dependent and time-dependent responses. Fish maintained at 20 kg/m^3^ showed the highest cortisol values across sampling points, whereas cortisol was also elevated in the 40 kg/m^3^ group at the end of the trial. At the molecular level, hepatic lep expression was strongly increased at the highest stocking density, while intestinal vip expression increased transiently at day 21 and returned towards baseline by day 40 [22]. These responses illustrate that endocrine and peptide-hormone markers can follow different temporal trajectories during prolonged density exposure.

In *Oncorhynchus mykiss*, acute overcrowding at 125 kg/m^3^ for 2 h increased *hsp70* expression relative to the 10 kg/m^3^ control condition [27]. Among the isoforms evaluated, *hsp70a_exon518* showed the clearest response, supporting its potential for detecting acute overcrowding stress in this species [27]. Longer-term density-related effects were also associated with reduced weight gain and growth, despite increased feed intake and oxygen consumption [3]. These changes were accompanied by lower plasma glucose and free amino acids, hepatic upregulation of *sod*, *cat*, *gst*, and *hsp90* and reduced expression of *ghr*, *igf1*, and *igf2*, suggesting an inhibition of the GH–IGF axis under stress conditions associated with overcrowding [3]. In white muscle, an upregulation of protein degradation-related genes (*murf1* and *fbx32*), along with increased *mtor* expression, was observed, suggesting an alteration in protein turnover [3]. Importantly, low stocking density may also act as a stressor in socially organised species. In *O. mykiss,* rearing at low densities (3.76 kg/m^3^) resulted in higher mortality rates (higher than those observed at high densities) and surviving individuals exhibited abnormalities in growth, feeding behaviour and physiology [13]. This low density prevented the establishment of a stable hierarchical system, leading to social stress and increased cortisol levels, although lactate and lysozyme remained unchanged [13].

Low-density conditions were also associated with reduced brain catecholamines and indoleamines, including L-DOPA and 5-HT, together with altered haematological and immune profiles characterised by increased neutrophils, reduced lymphocytes and upregulation of *irf-1β* and *irf9* at the lowest density relative to higher-density groups [13]. These findings illustrate that stocking density does not follow a simple linear relationship with welfare: both overcrowding and excessive social dispersion may be detrimental, depending on the behavioural ecology of the species.

For *Cilus gilberti* (Corvina drum), determination of optimal stocking density is crucial for aquaculture diversification. In this species, three different stocking densities were evaluated: 15, 25 and 35 kg/m^3^. All groups gained weight, but the increase was greatest at 35 kg/m^3^. Molecular analysis revealed that *igf1* levels were elevated in individuals reared at higher densities. Igf-1 plays a key role in somatic growth and muscle function, and its overexpression is associated with improved growth. By contrast, plasma glucose, hepatic glycogen, plasma and hepatic triacylglycerols, and Hsp70 did not differ significantly among groups, although Hsp70 tended to increase at high density [38]. These results show that a numerically higher density does not necessarily indicate greater stress when it remains within the species-specific optimal range.

In zebrafish (*Danio rerio*), exposure to high rearing densities (66 fish/L) from 18 dpf to 45 dpf can skew the sex ratio of the exposed population towards males, as occurs with temperature, although to a lesser extent [39]. Growth is inversely proportional to density and shows a sexual dimorphism in response to stress [39]. Two useful biomarkers for detecting the effects of high-density stress could be *dnmt1* and *cyp19a1a* genes [39]. The latter is a strong candidate due to its sensitivity to environmental stressors, particularly during the sexual differentiation phase, and it stands out as a marker of ovarian response to stress. Meanwhile, *dnmt1* represents a suitable epigenetic candidate, as it is altered in the ovaries almost two months after the end of treatment, showing hypomethylation [39].

In *Ompok bimaculatus* (Butter catfish) reared in a biofloc system, increasing stocking density elevated cortisol and glucose [40] and modulated genes involved in cellular stress, immune response and antioxidant defence, including *hsp70*, *nrf2*, *il-1b*, *il-20* and *tlr9* [40]. The coexistence of endocrine and transcriptional responses supports the presence of a detectable molecular signature of density-related stress in this production system.

Studies on *Sparus aurata* (Sea bream) confirm that stress assessment can be performed using conventional biomarkers; however, a more comprehensive approach involves integrating endocrine and metabolic markers [41]. After these animals were subjected to an overcrowding stress protocol, with a stocking density of 40 kg/m^3^, an increase in cortisol and decreases in glucose and triglyceride levels were observed. In addition, modulation of *crh* and *crhbp* was detected, confirming activation of the HPI axis [41]. Changes were also observed in the vasotocinergic and isotocinergic systems, particularly in their receptors (*avtrv1* and *avtrv2*) [41]. Thus, AVT, IT and their receptors may serve as valuable complementary biomarkers, given their strong dependence on tissue type and the nature of the stressor, which is especially relevant for welfare and management studies in the aquaculture industry [41].

Collectively, stocking density affects endocrine, metabolic, immune, growth-related, behavioural and epigenetic pathways. However, neither the direction nor the magnitude of these responses is consistent across species: similar nominal densities may represent overcrowding, an optimal production range or insufficient social contact depending on species biology, developmental stage and farming system. Cortisol remains a useful indicator of rapid endocrine activation, whereas HSPs, antioxidant and immune genes, miRNAs, growth-axis components and epigenetic markers provide complementary information on longer-term adaptation and physiological consequences. Accordingly, density-related welfare cannot be inferred from a universal numerical threshold or a single biomarker; it requires species-specific reference ranges and multimarker validation under relevant production conditions.

#### 4.2.3. Temperature

Temperature emerged as the most frequently investigated stressor among the studies included in this systematic review, reflecting its relevance to both aquaculture productivity and fish welfare. Its importance is further reinforced in the context of climate change [28,42,43], which is increasing the occurrence of extreme thermal events in aquatic environments [44]. Both unusually low temperatures [5,44] and progressive global warming [28,42] can challenge fish homeostasis. As ectothermic organisms, fish are particularly dependent on environmental temperature, which directly influences growth, feed efficiency, physiological function, immunity and oxidative status [7,14,45,46,47].

Within this context, molecular biomarkers provide a means of characterising not only the occurrence of thermal stress but also the mechanisms underlying the response in fish. Among the most consistently investigated are heat shock proteins, such as Hsp70, and components of the antioxidant defence system, such as superoxide dismutase (Sod), which reflect the activation of cellular protective and adaptive response mechanisms that contribute to survival and physiological homeostasis [7,48].

In milkfish (*Chanos chanos*), the response to cold is characterised by modulation of antioxidant defence systems, including changes in the expression of enzymes such as Sod1, catalase (Cat), glutathione peroxidase (GPx) and peroxiredoxin 6 (Prdx6). These alterations are accompanied by shifts in cellular redox status, reflected by hydrogen peroxide (H_2_O_2_) levels and by changes in cell viability, commonly assessed through apoptosis assays such as TUNEL. Together, these observations indicate that temperature stress affects not only gene expression and protein activity but also redox homeostasis and cell survival [44]. These responses illustrate how molecular and cellular endpoints can provide complementary information on the disruption of redox homeostasis during hypothermal stress. Cold exposure also affects the expression of warm-temperature-acclimation-associated protein 65 (Wap65). Two isoforms, *Ccwap65-1* and *Ccwap65-2*, have been identified in the liver, showing differential responses to hypothermal stress. In particular, *Ccwap65-2* has been associated with the immune response and cold-associated infection, with its expression also being influenced by ambient salinity [46].

The relevance of metabolic responses to cold exposure is also evident in other cold-sensitive species. In *Nibea albiflora* (Yellow drum), an economically important species in China, Japan and Korea which is highly sensitive to cold (with a reported lethal temperature of approximately 5 °C), the effects of low temperature were evaluated using a 14-day overwintering protocol in which fish were maintained under fed or fasted conditions at either 16 °C or 8 °C [5]. Metabolomic analysis revealed alterations in hepatic glutathione metabolism, with glutamate and oxidised glutathione (GSSG) proposed as potential biomarkers of overwintering stress after a 14-day protocol that compared fed and fasted groups at 16 °C (control) and 8 °C (cold). Their response was maintained despite differences in nutritional status. The consistent response of both metabolites across feeding conditions strengthens their potential as indicators of cold-related metabolic disturbance [5].

Beyond metabolomic responses, proteomic approaches have revealed additional levels of thermal adaptation. In common carp (*Cyprinus carpio*), acclimation to cold (10 °C) and warm (30 °C) induces clear differential expression of blood plasma proteins including Wap65, Cap31, haptoglobin, apolipoproteins and complement components [45]. Importantly, temperature-dependent differences were also detected among specific proteoforms, indicating that molecular variants of the same protein may provide additional discriminatory information beyond total protein abundance [45].

Biomarker panels have also been used to evaluate strategies aimed at mitigating thermal stress. In a separate study, *C. carpio* were supplemented with *Lactobacillus helveticus* and chlorogenic acid for eight weeks and subsequently exposed to a thermal stress protocol ranging from 25 °C to 32 °C [49]. The intervention was associated with changes in immune parameters, antioxidant defences and serum biochemical/stress indicators, including increases in lysozyme, ACH50, immunoglobulins, Cat, Gpx and Sod, together with decreased MDA, cortisol and glucose [49]. Together, these changes reflect comprehensive improvements: lysozyme and Ig enhance immune function; Cat, GPx and Sod counteract oxidative stress, whereas lower MDA, cortisol, glucose and liver enzymes indicate reduced physiological stress [49]. These biomarkers, easily measured using standard, routine kits, are well suited for monitoring welfare and productivity in intensive aquaculture [49].

The diversity of molecular approaches used to characterise thermal stress is particularly evident in salmonids, where studies range from targeted proteomics to transcriptional profiling under both warming and cooling conditions. In *Salmo salar*, targeted proteomic approaches have enabled the identification and validation of heat-responsive peptides derived from proteins including SerpinH1, elastase 2 (Ela2) and glyceraldehyde-3-phosphate dehydrogenase (Gapdh). These peptide signatures reflect changes in protein abundance and/or processing under thermal stress, supporting their utility as molecular indicators of heat-induced physiological disruption [42]. Among these, SerpinH1 is particularly noteworthy because of its recurrent association with stress responses in salmonids and its involvement in collagen metabolism, linking the cellular stress response with traits of direct aquaculture relevance, such as flesh texture [42]. These findings illustrate the potential of targeted proteomics for biomarker identification and validation, in this case in salmon liver, and support its application in monitoring heat stress mitigation [42].

Beyond targeted peptide signatures, gradual warming in Atlantic salmon induces a broader transcriptional response, particularly when combined with additional environmental challenges. Under gradual warming, either alone or combined with moderate hypoxia, *S. salar* activates a broad cellular defence response [50]. SerpinH1, Hsp70 and Hsp90 are among the most prominent molecular markers, with *serpinh1*, *hsp90aa1* and *hsp70* showing increased expression in the liver during progressive warming [50,51]. Their coordinated response reflects the activation of mechanisms involved in protein homeostasis and cellular protection during thermal challenge.

Thermal exposure also affected genes linked to innate and adaptive immunity, a finding that is particularly important in aquaculture due to its implications for susceptibility to infections [50]. The activation of immune-related genes, including *c1ql2*, *casp8*, *tnfrsf6b*, *apod*, *epx*, *camp-a* and *il8*, indicates that elevated temperature can reshape constitutive immune status, reinforcing the multifunctional nature of the response [51]. In parallel, a downregulation of *hif1α* and *egln2*, together with that of oxidative stress- and mitochondrial-associated genes such as *cyp1a1*, *ucp2*, *prdx6*, *cirbp* and *rraga*, points to a coordinated reprogramming of redox balance and energy metabolism under sustained thermal challenge [51]. Taken together, the downregulation of *cyp1a1*, *ucp2*, *prdx6* and *cirbp* reinforces the idea of a partially suppressed cellular response to prolonged exposure to high temperature and hypoxia [50]. Importantly, the lack of clear additive effects under combined heat and moderate hypoxia suggests that these stressors converge on overlapping pathways rather than acting independently [50]. This finding also illustrates a limitation of individual biomarkers when fish are exposed to multiple simultaneous challenges and supports the use of integrated molecular profiles [51].

Importantly, the molecular response of *S. salar* is not restricted to warming, as progressive cooling also elicits a distinct stress signature. Gradual cooling in *S. salar* induces progressive physiological changes associated with stress, affecting osmoregulation, metabolism and liver function. In a protocol involving a temperature decrease of 1 °C per week from 8 to 1 °C, plasma cortisol did not increase until fish reached approximately 1–2 °C, whereas an earlier increase in plasma glucose between 8 and 6 °C was considered likely to reflect differences in feeding and sampling time rather than a specific cold-stress effect [52]. Furthermore, *hsp70b*, *hsp70c*, *hsp90aa1a* and *hsp90aa1b* were among the strongest candidate molecular biomarkers during cooling. Importantly, changes in *hsp* transcript expression began at higher temperatures than the cortisol response, suggesting that these molecular markers may provide earlier indications of progressive cold stress than plasma cortisol [52]. Whereas these studies focused largely on individual candidate genes, transcriptomic approaches allow thermal responses to be interpreted within broader multigene signatures.

Moving from individual candidate genes towards multigene signatures, transcriptomic approaches in *Oncorhynchus kisutch* (Coho salmon) have been used to discriminate responses to temperature (10, 14 and 18 °C), salinity (0, 20 and 28 ppt) and hypoxia [43]. In the case of temperature, consistent gill markers included the Serpin family genes *TM_SERPIN20_379* and *TM_SERPIN9_380* [43]. Their inclusion within broader molecular classifiers illustrates the potential advantage of gene panels when the objective is to distinguish among multiple environmental stressors rather than merely detect a general stress response.

In addition to the type of molecular approach used, the developmental stage at which thermal exposure occurs can substantially influence the subsequent biomarker response. In rainbow trout (*Oncorhynchus mykiss*), thermal challenge during early development characterised *hsp70a* and *hsp70b* as the main biomarkers of interest [53]. These data suggest that early thermal shock can partially alter the subsequent transcriptional response of the *hsp70* family to heat, particularly that of *hsp70a* [53]. Although embryo survival was unaffected up to 6 days post-hatching, body weight was reduced at this stage [53]. These findings indicate that early thermal exposure can produce persistent molecular and phenotypic effects that would not be captured by survival alone.

A similar need for biological context is evident when biomarker responses are considered across different tissues and developmental stages. The importance of integrating molecular responses with tissue-level information is illustrated in *Dicentrarchus labrax*. Various biomarkers have been established to assess heat stress, combining molecular and histological approaches that enable the detection of physiological responses together with their tissue localisation [35]. Immunohistochemical studies using Hsp70, MDA, NT, and 4-HNE have enabled the identification of the presence and distribution of stress-associated markers in organs such as the kidney, liver, spleen and gills [35], while *hsp70* is also overexpressed at the molecular level under thermal challenge [54]. Furthermore, the observation of rodlet cells following exposure to stressors supports their potential value as a novel complementary indicator of distress or adverse conditions [35]. In parallel, *nr3c1* and *nr3c2*, encoding glucocorticoid and mineralocorticoid receptors linked to stress response and energy mobilisation, showed stage-dependent regulation under thermal challenge [28]. Together, these observations emphasise that both tissue and developmental stage can substantially influence biomarker interpretation.

Environmental stressors may also interact, further complicating the interpretation of individual biomarkers. In industrial settings, thermal stress is often accompanied by changes in salinity, so the interaction between these stressors is relevant [54]. This combination elicits environment-dependent physiological and molecular responses: *Dicentrarchus labrax* maintained at intermediate salinities (6 and 12 psu) showed better growth and less biochemical disturbance, whereas those at extreme salinities (32 and 2 psu) exhibited higher stress levels, reflected by increased cortisol and decreased glucose and triglycerides [54]. At the molecular level, alongside Hsp70 overexpression, reduced Igf-1 expression in the kidney and gills and alterations in osmoregulatory genes such as *Na+/K+ ATPase α1*, *nkcc1* and *cftr* further support a coordinated response to combined thermal and osmotic stress [54]. The coordinated changes across endocrine, metabolic, stress-response and osmoregulatory pathways illustrate how the biomarker profile associated with thermal challenge may vary depending on concurrent environmental conditions such as salinity [54].

Across other teleost species, HSPs remain among the most recurrent thermal-response markers, although their behaviour varies according to species, tissue and exposure duration. In *Perca flavescens* (yellow perch), *hsp70* showed its strongest response at 26 °C, whereas *igf1* displayed an inverse pattern and antioxidant markers such as Sod, GPx, and Gsr responded more variably to temperature and salinity [4].

In *Micropterus salmoides* (largemouth bass), cortisol and blood glucose increased after both acute and chronic heat exposure, whereas lactate increased only after acute exposure [55]. The different temporal profiles again highlight exposure duration as a critical factor in biomarker interpretation [55].

Studies on *Sander lucioperca* investigated antioxidant-defence genes, including *sod*, *cat*, *gpx*, and *gr*, together with *hsp70* and *hsp90* [7]. Their combined analysis therefore captures complementary antioxidant and cellular-protection responses [7].

In *Megalobrama amblycephala* (Blunt snout bream), Hsp90b showed a faster and more sustained induction than Hsp60 [6], further demonstrating that biomarkers within the same broad stress-response system may differ in temporal sensitivity.

In *Labeo rohita* muscle tissue, *Serpine1* (*Hsp47*) showed a consistent induction under heat stress, whereas *Hsp70* and *Hsp90* responses varied with tissue and experimental conditions [56]. PGC-1α, myozenin-2, proto-oncogene c-Fos and acetyl-CoA carboxylases (ACCs) were also identified as candidates, albeit to a lesser extent [56].

The variability observed among classical stress-response genes becomes even more evident when thermal extremes are examined across different regulatory levels. In Nile tilapia (*Oreochromis niloticus*), *Hsp70* showed elevated levels in liver and gill tissues without seasonal differences, while *hsp27* was upregulated in summer with higher expression in liver than gills [57]. Gst and immune-related genes such as *tlr*, *il-1* and *il-8* also displayed seasonal responses, while both hot and cold conditions were associated with DNA fragmentation and micronucleus formation [57]. These results illustrate that opposite thermal extremes may share some cellular consequences while retaining distinct molecular signatures.

At a different regulatory level, miRNAs have been investigated as potential indicators of cold tolerance in this species. Their relevance, however, appears to depend on the tissue studied and on whether they reflect a pre-existing state of tolerance or a response induced by cold exposure. miR-122 and miR-92a distinguished cold-tolerant from cold-sensitive individuals prior to thermal exposure, whereas miR-30b reflected the response following the cold challenge [58]. In brain tissue, miR-9-5p and miR-9-3p were elevated in cold tolerant fish after CTmin test, while miR-135c did not differ from the unstressed control but did differ between cold-tolerant and cold-sensitive fish [58]. These findings highlight an important distinction between pre-existing molecular signatures associated with cold tolerance and biomarkers induced by thermal exposure, as these two types of markers provide different information for stress assessment.

Beyond the more extensively studied HSP families, alternative molecular markers may provide information on specific components of thermal damage. A more recent marker for assessing heat stress in teleosts is UNC-45B, a myosin chaperone involved in the refolding of heat-denatured myosin [59]. In Pacific bluefin tuna (*Thunnus orientalis*), UNC-45B expression increased significantly in summer (at both transcriptional and translational levels), showing a response to heat shock [59]. In particular, isoform 2 exhibited greater stability and resistance to degradation, reinforcing its role in the response to heat stress [59]. Its close relationship with myosin homeostasis makes UNC-45B particularly relevant as a candidate indicator of muscle-specific responses to heat, complementing broader cellular stress markers [59].

Finally, genomic approaches extend the concept of thermal biomarkers from indicators of exposure towards potential predictors of inherent tolerance. In *Esox lucius*, 77,617 SNPs were identified using a GBS approach. Subsequent GWAS analysis revealed nine key SNPs associated with heat tolerance and cumulative survival time [60]. Based on these loci, four candidate genes related to thermal tolerance were identified: *clstn2*, *htr4*, *atp13a3*, and *schip1* [60]. These findings support the polygenic nature of thermal tolerance and illustrate the potential of genomic markers for identifying resilient phenotypes [60].

Taken together, the studies reviewed show that thermal stress cannot be characterised by a single molecular response. HSPs and antioxidant pathways are among the most recurrent indicators, but their magnitude and temporal profiles vary substantially with species, tissue, developmental stage, exposure duration and the presence of additional environmental stressors. Proteomic, miRNA, transcriptomic and genomic approaches further expand the information obtained, ranging from the detection of cellular stress and acclimation to the identification of pre-existing signatures of thermal tolerance. Thus, rather than identifying a universal thermal biomarker, the available evidence supports the selection of complementary markers according to the biological context and the specific objective of stress assessment.

#### 4.2.4. Other Stressors

Although temperature, stocking density, handling and transport accounted for the most extensively represented stressors in the studies reviewed, other aquaculture-relevant challenges were also identified. These included hypoxia, salinity changes, nutritional stress, chemical exposure and other environmental or husbandry-related conditions. Because these stressors were represented by fewer or more heterogeneous studies, their associated species and biomarkers are summarised in Table 2 rather than discussed individually.

Several limitations should be considered when interpreting the findings of this systematic review. First, the review was deliberately restricted to studies in which molecular biomarkers constituted a primary component of stress assessment. Studies evaluating exclusively physiological, biochemical or behavioural indicators were therefore excluded, although conventional physiological indicators such as cortisol were considered when analysed alongside molecular endpoints. This approach allowed the review to maintain a clear focus on molecular stress assessment but necessarily excluded part of the broader literature on fish stress and welfare.

Second, substantial methodological heterogeneity among studies—including differences in species, stress protocols, exposure duration, biological matrices, sampling time and analytical techniques—limited direct comparisons and precluded a meaningful quantitative synthesis. This heterogeneity also complicates the establishment of common reference ranges or universal biomarker thresholds across aquaculture species.

An additional limitation is that no formal post-inclusion risk-of-bias assessment was performed for the 64 studies included in the qualitative synthesis. Although all included studies met the predefined methodological quality and aquaculture applicability threshold during full-text eligibility assessment, this screening procedure was not intended to replace a formal risk-of-bias assessment. Some variation in methodological quality may therefore remain among the included studies and should be considered when interpreting the strength of the synthesised evidence.

Another limitation is that six potentially relevant studies could not be assessed because their full texts could not be retrieved after reasonable attempts. Although this represents only a small proportion of the records evaluated, their exclusion constitutes a potential source of selection bias.

A further limitation concerns the translation of experimental findings to commercial aquaculture. Although some studies reproduced aspects of routine farming practice, including commercial transport or production-scale rearing conditions, most retained a substantial degree of experimental control. Such designs cannot fully reproduce the complexity of commercial environments, where fish may be simultaneously exposed to interacting environmental, husbandry and biological challenges. Consequently, the sensitivity, specificity and temporal stability of many candidate molecular biomarkers remain to be established under routine farming conditions before their implementation in welfare-monitoring programmes. Practical implementation will also depend on sampling burden, analytical cost and turnaround time, equipment requirements and the availability of trained personnel, which vary substantially among biomarker platforms.

These limitations define the main challenges for translating molecular biomarkers from experimental research into practical aquaculture tools. Future progress will depend less on identifying an increasing number of candidate markers than on validating the most informative ones across biological contexts, standardising analytical approaches and establishing their performance under commercial conditions. In this framework, molecular biomarkers are best viewed as complementary components of integrated welfare assessment rather than as replacements for established physiological indicators.

## 5. Conclusions

This systematic review synthesised the evidence published over the last decade on molecular biomarkers used to characterise stress responses in teleost species relevant to aquaculture. Temperature was the most extensively investigated stressor, followed by husbandry-related challenges such as stocking density, handling and transport. Across these conditions, the evidence consistently demonstrates that stress responses are multifactorial and highly context-dependent, involving coordinated endocrine, cellular, metabolic, oxidative and immune mechanisms whose magnitude and temporal dynamics vary among species and experimental conditions.

No single biomarker emerged as universally informative across all stressors and biological contexts. Cortisol remains a widely used indicator of endocrine stress activation, but its interpretation depends strongly on the biological matrix, sampling time and the acute or chronic nature of the challenge. HSPs, particularly Hsp70 and Hsp90, provide complementary information on cellular stress responses, although their expression is similarly influenced by species, tissue and exposure conditions. miRNAs and broader omics-derived signatures expand this framework by capturing regulatory responses and, in some cases, molecular profiles associated with stress tolerance or resilience.

Overall, the evidence supports a shift from the search for universal single biomarkers towards integrated, context-specific panels combining molecular and established physiological indicators. The principal challenge is now to determine which combinations provide reproducible and biologically meaningful information across species, stress scenarios and sampling conditions. Standardisation of analytical approaches, definition of appropriate reference ranges and validation under commercial farming conditions will therefore be essential for translating molecular biomarker research into reliable tools for routine welfare assessment in aquaculture.

## Figures and Tables

**Figure 1:**
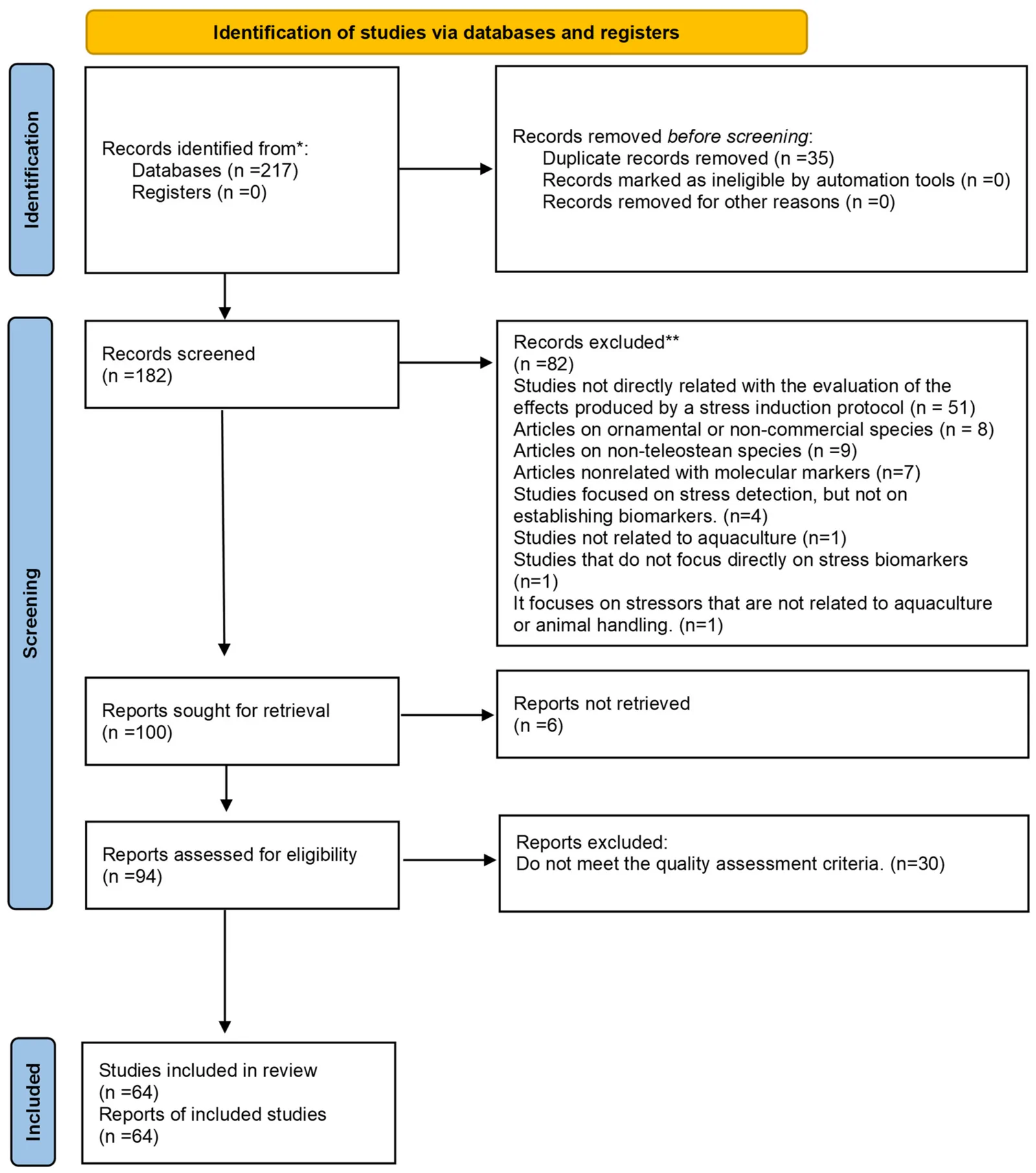
PRISMA 2020 flow diagram illustrating the study selection process. A total of 217 records were identified through database searching. Following duplicate removal and eligibility assessment, 64 studies were included in the qualitative synthesis. At the full-text eligibility stage, studies scoring below the predefined methodological quality and applicability cut-off of 10.5/12 were excluded; the complete assessment criteria and scoring system are provided in Supplementary Table S2. * Records were identified within the predefined search period (2015–13 June 2025). ** Records were excluded according to the predefined eligibility criteria described in Table 1.

**Figure 2:**
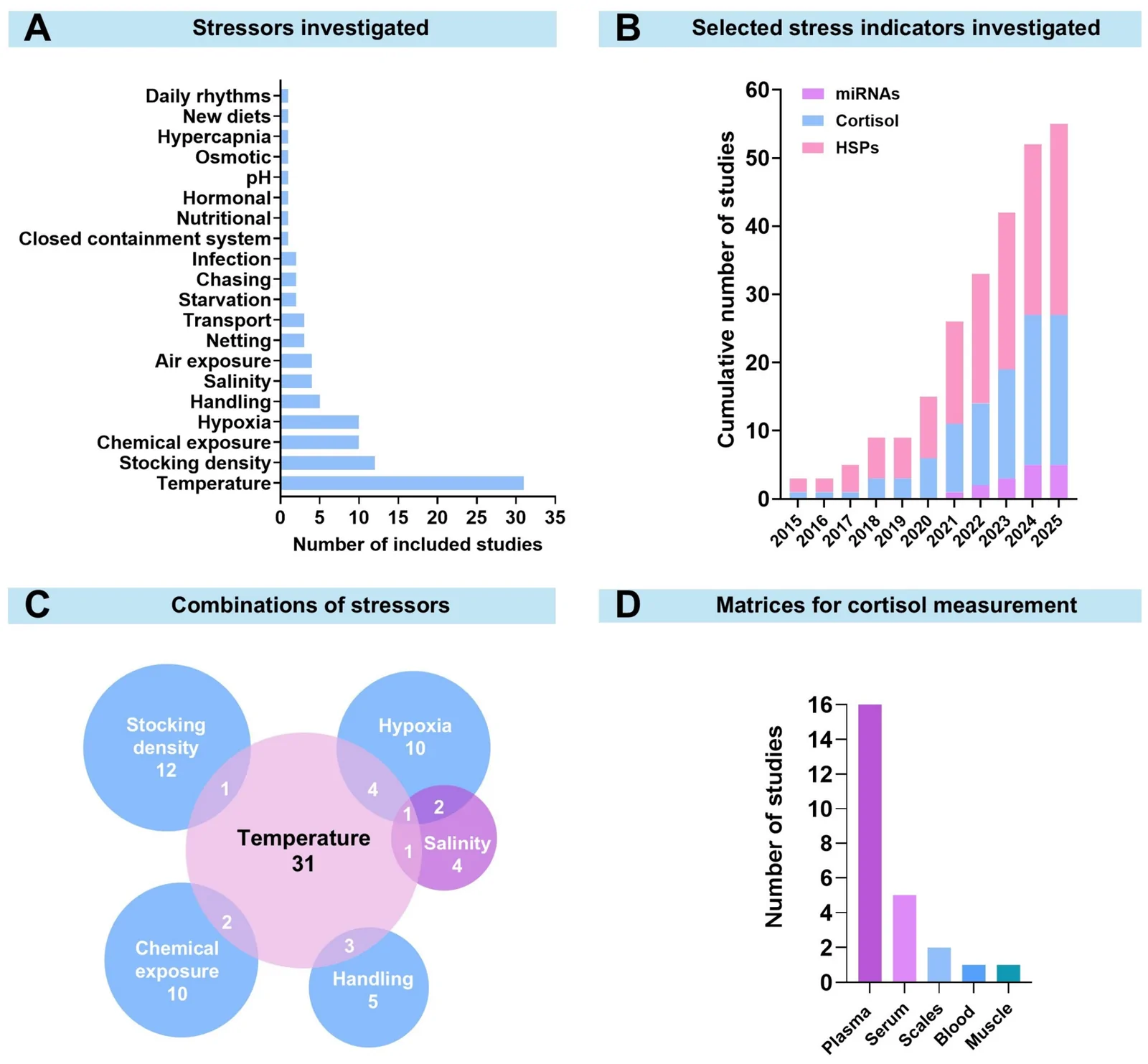
(**A**) Number of included studies evaluating each stressor. Studies evaluating more than one stressor were counted in each corresponding category. (**B**) Cumulative number of included studies evaluating the selected stress indicators over time. Studies evaluating more than one stress indicator were counted in each corresponding category. (**C**) Combinations among the most frequently investigated stressors. Labels within each stressor indicate the total number of studies evaluating that stressor, either alone or in combination, whereas values in overlapping areas indicate the number of studies evaluating the corresponding combinations of stressors. (**D**) Biological matrices used for cortisol quantification in the included studies.

**Figure 3:**
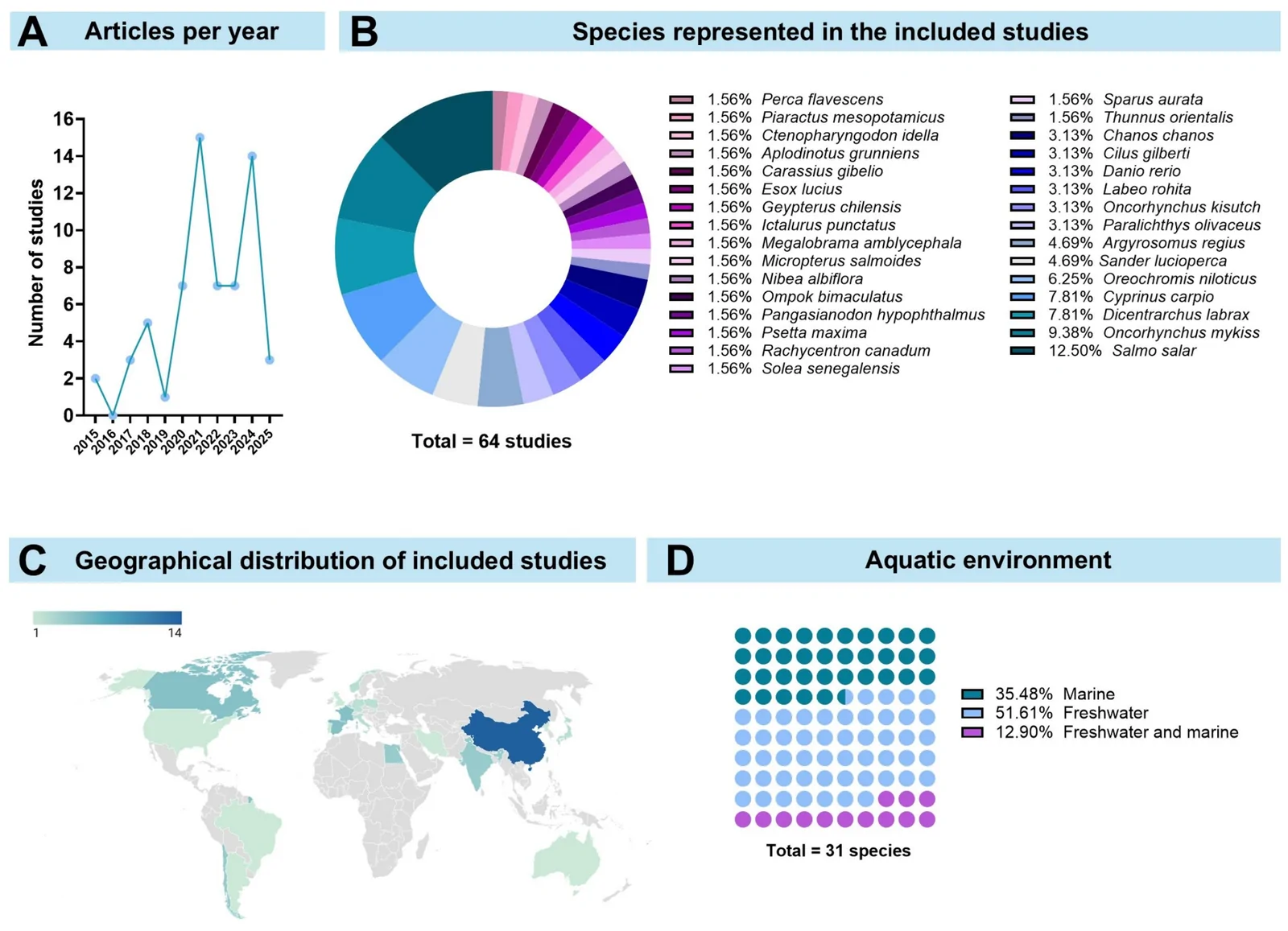
(**A**) Number of included studies published per year from 2015 to 13 June 2025. Note that 2025 represents a partial year because the final database search was conducted on 13 June 2025. (**B**) Frequency of teleost species represented in the 64 included studies; percentages refer to the proportion of included studies in which each species was investigated. (**C**) Geographical distribution of the included studies according to the country classification described in the Methods. (**D**) Distribution of the 31 represented species according to their aquatic environment: freshwater, marine, or both freshwater and marine. Each dot represents approximately 1% of the 31 species represented.

**Table 1:** Representation of inclusion and exclusion criteria in the first phase of the PRISMA method.

Criterion	Inclusion	Exclusion
Date	2015–13 June 2025	Prior to 2015
Language	Articles in English	Articles not available in English
Industry	Aquaculture	Other industries
Species	Teleost	Non-teleost species
Type of teleosts	Aquaculture species or established experimental model species	Non-aquaculture species without established model relevance
Stressors	Related to aquaculture or animal husbandry	Unrelated to the aquaculture sector
Markers	Molecular biomarkers, alone or with complementary physiological indicators	Studies without molecular biomarker assessment
Stress detection	Studies evaluating molecular biomarkers in relation to a defined stress condition	Studies not evaluating molecular biomarkers in relation to a defined stress condition.
Focus	Stress biomarkers	Biomarkers not related to stress
Protocols	Stress induction protocols	No stress induction protocols
Parasitic infection	Parasitic infection evaluated together with another aquaculture-relevant stressor	Parasitic infection as the sole experimental stressor
Full-text availability	Full-text available for eligibility assessment	Full-text unavailable after retrieval attempts

**Table 2:** Studies included in the systematic review addressing stressors not discussed individually in the main text, together with the teleost species and biomarkers evaluated. Note: A single reference may appear in more than one stress category when a single study assessed different stress factors or experimental conditions. When studies assessed the combined effect of multiple stress factors (i.e., more than two simultaneous stress factors), they were classified under the ‘Combined’ category.

Stressor	Species	Marker	Ref
Administration of oxytetracycline	*Oncorhynchus kisutch*	ALT, AST, GSH, GPX, GR, LPO, HSP70, HSI	[8]
Air exposure	*Dicentrarchus labrax*	Cortisol–Glucose–Lactate–*crh*–*crh-bp*–cat–sod1–gsh-px–gsr–ucp1–coxIV–prdx3	[37]
	*Oncorhynchus mykiss*	ssa-miR-16b-5p, ssa-miR-30b-5p, ssa-miR-26a-5p, miR-16b-5p and miR-16c-5p.	[33]
Nutritional stress	*Sander lucioperca*	*StAR*, *Gr*, *Ppara*, *Hsl*, *Pepck*, *pge2*, *pla2*, *cox2*, *5-lox*, *gpx*, *sod*, *cat*, *alp*, *twist2*, *i-fabp*	[61]
Avermectin exposure	*Cyprinus carpio*	ROS, MDA, CAT, GSH, T-AOC, *sod*, *gpx*, Nrf2, Keap1, *tnf*-α, *il-6*, *inos*, il-1β, *tgf*-β1, *il-10*, IκBα, P-NFκB-p65, *grp78*, *atf6*, *ire1*, *chop*, *perk*, *eif2a*, LC3-II, ATG5, PINK1, PARKIN, BNIP3	[62]
Cadmium exposure	*Carassius gibelio*	*hsp70, nrf2, mt, atf6, bip, xbp1, eif2α, atf4, chop, beclin1, atg5, lc3b, bax, bcl2, casp9* and *casp3*.	[63]
	*Paralichthys olivaceus*	*hsp70, mt, lzm* and *hsp110*.	[64]
Change in Growing System	*Salmo salar*	cortisol–glucose–magnesium–keratin, type I cytoskeletal 20–epithelial membrane protein 3–thrombospondin-2–cadhl, *emp3*, and thbs2–heat shock protein 90–mucin 5b–mucin 2	[65]
Combined	*Cyprinus carpio* L.	Cortisol, *crf*, *pomc*, *star* and ATP1a1a	[26]
	*Danio rerio*	miR-29a	[14]
	*Pangasianodon hypophthalmus*	Cortisol, inducible nitric oxide synthase (iNOS), heat shock protein (HSP70), and metallothionein (MT), catalase (CAT), superoxide dismutase (SOD), and glutathione peroxidase (GPx)–caspase 3a (CAS 3a), tumour necrosis factor (TNFα), interleukin (IL), and toll-like receptors (TLR)	[66]
Enrofloxacin exposure	*Ctenopharyngodon idella*	SOD, CAT, GSH, MDA, ACHE, NO, IBR, Amylase and lipase	[67]
Hormonal induction	*Sander lucioperca*	Cortisol, glucose, hamp, testosterone, *tnf*-α, *il-1, lys, c3*, ACH50, peroxidase, lysozyme	[68]
Hypercapnia	*Psetta maxima*	IL-1b, LMP7, Grim19, PRDX, Akirin, STAT2, COX, EF1α, GST and DFAD	[69]
Hypoxia	*Cilus gilberti*	cgLep, cgLepR, NPY	[9]
	*Oncorhynchus mykiss*	miR-122-5p, miR-184-3p, miR-194a-5p, miR-210-3p, miR-218c-5p, miR-153a-3p	[70]
	*Oncorhynchus mykiss*	HSP70a, HSP70a exon 518, HSPA4, HSPA4L, HSPA14, HSPA8/HSC70, HSC70b, HSC71	[27]
	*Oreochromis niloticus*	Il8, Il-1B, Ifn-y–Tnf-a, Caspase 3 and Il-10	[71]
	*Rachycentron canadum*	Composition and diversity of the microbiota	[72]
Methamidophos exposure	*Paralichthys olivaceus*	HSP90, GzmK, ASL	[73]
pH	*Paralichthys olivaceus*	*hsp70, mt, lzm* and *hsp110*.	[64]
Prolonged exposure to hydrogen sulphide	*Salmo salar*	Cortisol, lactate, glucose, *cat*, *gpx*, cu/zn-*sod*, *sqor2*, *cyp450*, *hsp90*, *bax*, *casp3a*, granz, *cd4*, *il-8,* *il-12, igt, sigm, muc2, muc5b*	[74]
Salinity	*Aplodinotus grunniens*	HSP70, HSP90, SOD, CAT, MDA, TAOC, ALT, AST and Na+/K+-ATPase	[75]
	*Oreochromis niloticus*	Il8–Il-1B–Ifn-y–Tnf-a–Caspase 3–Il-10	[71]
Triphenyltin exposure	*Cyprinus carpio*	SOD, CAT, GPx, GSH, MDA, NO, Hsp70, Hsp90, lysozyme,	[76]

## Data Availability

No new datasets were generated. All information analysed in this systematic review was derived from the cited publications and the Supplementary Materials.

## Supplementary Materials

The following supporting information can be downloaded at: https://www.mdpi.com/article/10.3390/biology15191681/s1, Table S1: The search strategy was designed and managed within the Parsifal platform. The same keyword combination was applied across all databases, with syntax adapted by the platform where appropriate.; Table S2: Predefined methodological quality and applicability assessment criteria used during full-text eligibility assessment. Each criterion was scored as Yes (1 point), Partially (0.5 points), or No (0 points). The maximum possible score was 12, and studies were required to achieve a predefined minimum score of 10.5 to be eligible for inclusion.; Table S3: Summary of the principal characteristics of the studies included in this systematic review, including the teleost species investigated, stressor(s), stress biomarkers evaluated, stress duration, biological matrices analysed and the corresponding DOI.

## Abbreviations

The following abbreviations are used in this manuscript:

8-OHdG	8-hydroxy-2′-deoxyguanosine
ACCs	Acetyl-CoA carboxylases
ACH50	Alternative complement haemolytic activity
ACHE	Acetylcholinesterase
ALP	Alkaline phosphatase
ALT	Alanine aminotransferase
AST	Aspartate aminotransferase
AVT	Arginine vasotocin
BPG	Brain-pituitary-gonadal axis
Cat	Catalase
CNS	Central nervous system
GBS	Genotyping-by-sequencing
GPx	Glutathione peroxidase
Gpt	Glutamate pyruvate transaminase
GSSG	Oxidised glutathione
GWAS	Genome-wide association study
HNE/4-HNE	4-hydroxynonenal
HPI	Hypothalamic-pituitary-interrenal axis
HSPs	Heat shock proteins
IBR	Integrated Biological Response index
IT	Isotocin
Ldh	Lactate dehydrogenase
MDA	Malondialdehyde
Mdh	Malate dehydrogenase
miRNAs	microRNAs
Mt	Metallothionein
ncRNAs	Non-coding RNAs
NO	Nitric oxide
NT	Nitrotyrosine
PRISMA	Preferred Reporting Items for Systematic Reviews and Meta-Analyses
ROS	Reactive Oxygen Species
SLR	Systematic Literature Review
SNPs	Single nucleotide polymorphisms
SOD	Superoxide dismutase
T-AOC	Total antioxidant capacity
TUNEL	Terminal deoxynucleotidyl transferase dUTP nick end labelling
UNC-45B	Uncoordinate-45B (myosin chaperone)
Wap65	Warm-temperature-acclimation-associated 65-kDa protein
