# Supplementary material for: Molecular Stress Biomarkers in Aquaculture: A Systematic Review
Source: Biology (Basel). 2026 Sep 22;15(19):1681. doi: 10.3390/biology15191681 (PMC13663503; doi:10.3390/biology15191681)
Supplement: Supplementary file 1 [file biology-15-01681-s001.zip › biology-4424701-supplementary.pdf]

Molecular stress biomarkers in aquaculture: A systematic review

Alba Sellés-Egea [1], David G. Valcarce [1] and Vanesa Robles [1,*]

Supplemetary Material

Table S1. The search strategy was designed and managed within the Parsifal platform. The same keyword combination was applied across all databases, with syntax adapted by the platform where appropriate.

Data bases	Data of the last search	Time period	Language	Search string
Web of Science	13.June.2025	2015 - 2025	English	TS=("stress response" OR "physiological stress") AND TS=("molecular markers" OR biomarkers OR "stress biomarkers") AND TS=(fish OR teleost) AND TS=(aquaculture OR "fish farming") AND TS=("gene expression" OR transcriptomics)
Scopus	13.June.2025	2015 - 2025	English	TS=("stress response" OR "physiological stress") AND TS=("molecular markers" OR biomarkers OR "stress biomarkers") AND TS=(fish OR teleost) AND TS=(aquaculture OR "fish farming") AND TS=("gene expression" OR transcriptomics)
PubMed	13.June.2025	2015 - 2025	English	("stress response" **OR** "physiological stress") **AND** ("molecular markers" **OR** biomarkers **OR** "stress biomarkers") **AND** (fish **OR** teleost) **AND** ("aquaculture" **OR** "fish farming") **AND** ("gene expression" **OR** transcriptomics)

Table S2. Predefined methodological quality and applicability assessment criteria used during full-text eligibility assessment. Each criterion was scored as Yes (1 point), Partially (0.5 points), or No (0 points). The maximum possible score was 12, and studies were required to achieve a predefined minimum score of 10.5 to be eligible for inclusion.

Criterion	Assessment question
Molecular biomarker	Is any molecular stress marker analyzed?
Stress response	Does it precisely and adequately analyze the effect produced by stress?
Species relevance	Is the species under study relevant in aquaculture or is it a model species with potential translational value?
Stress protocol	Does it adequately describe the type of stress or combination of stressors?
Stress quantification	Is stress quantified in the study?
Farming conditions	Are the cultivation conditions representative of aquaculture practice?
Industrial relevance	Does the study have potential applicability to aquaculture?
Peer review	Is the article published in a peer-reviewed journal?
Control group	Does the study include an appropriate control group?
Aquaculture relevance	Are the stressors directly related to aquaculture?
Environmental realism	Are stress levels representative of farming conditions?
Complementary variables	Are molecular biomarkers complemented by physiological or health-related variables?

Table S3. Summary of the principal characteristics of the studies included in this systematic review, including the teleost species investigated, stressor(s), stress biomarkers evaluated, stress duration, biological matrices analysed and the corresponding DOI.

Article	Study species	Stressor	Biomarkers evaluated	Acute/Chronic	Study in	DOI
A multi-biomarker approach to evaluate the effect of sodium chloride in alleviating the long-term transportation stress of *Labeo rohita* fingerlings	*Labeo rohita*	Long term transportation	cortisol - sgpte -hsp70 - trigliceride -creatinine activity.	Acute		https://doi.org/10.1016/j.aquaculture.2020.735979
Acclimation to cold and warm temperatures is associated with differential expression of male carp blood proteins involved in acute phase and stress responses, and lipid metabolism.	*Cyprinus carpio L*	Cold and Warm temperatures	Cap31 - Wp65 - hemopexin-like	Acute	Inmune system	https://doi.org/10.1016/j.fsi.2018.03.018
Rapid detection of heat stress biomarkers in Atlantic salmon (*Salmo salar*) liver using targeted proteomics	*Salmo salar*	Temperature (heat stress)	beta-galactosidase_hprt1-HYADDLDR_pinh1a_ADLSNISGK-SerpinH1_TNSILFIGR-ela2_VVGGEDVR-gapdh_VPTPNVSVVDLTVR	Chronic	Liver	https://doi.org/10.1002/aff2.147
Characterization and gene expression profiles of *Cilus gilberti* leptin in response to culture parameters	*Cilus gilberti*	Acute hypoxia	Leptin	Acute/Chronic	Tissue samples (including spleen, gills, brain, pyloric caeca, stomach, liver, foregut, hindgut, white	https://doi.org/10.1016/j.aqrep.2024.102273

					muscle, anterior kidney and posterior kidney)	
Circulating MicroRNAs Indicative of Sex and Stress in the European Seabass (*Dicentrarchus labrax*): Toward the Identification of New Biomarkers	*Dicentrarchus labrax*	High density	Cortisol - miR-7132a-5p - miR-499a-5p - miR-1388-3p	Acute	Blood samples	10.1007/s10126-023-10237-0
Daily rhythms of acute stress responses and antioxidant systems in the European sea bass (*Dicentrarchus labrax*): Effects of the time of the year	*Dicentrarchus labrax*	Daily rhythms (estaciones) Air exposure	Cortisol - glucose - lactic acid - mRNA crh - cat - Sod1 - gshpx - gsr -	Acute	Plasma and hypothalamus - liver	https://doi.org/10.1016/j.aquaculture.2024.740616
Developing molecular classifiers to detect environmental stressors, smolt stages and morbidity in coho salmon, *Oncorhynchus kisutch*	*Oncorhynchus kisutch*	Temperature and normoxia (hypoxia) salinity	TABLE	Chronic		https://doi.org/10.1016/j.scitotenv.2024.175626
Development of molecular and histological methods to evaluate stress oxidative biomarkers in sea bass	*Dicentrarchus labrax*	Manipulation and oxygen concentration and temperature	hsp70	Acute	Histology and immunohistochemistry	10.1007/s10695-020-00811-x

(Dicentrarchus labrax)						
Dietary DHA and ARA level and ratio affect the occurrence of skeletal anomalies in pikeperch larvae (*Sander lucioperca*) through a regulation of immunity and stress related gene expression	*Sander lucioperca*	Experimental diets	pparα, fadsd6, elovl5, pepck, hsl, gr, StAR, pge2, pla2, lta4h, cox2, 5-lox, gpx, sod, cat, twist, mef2c, sox9, alp, and intestinal fatty-acid binding protein (i-fabp),	Acute	Larvae	https://doi.org/10.1016/j.aquaculture.2021.737060
Dietary sodium butyrate ameliorated the blood stress biomarkers, heat shock proteins, and immune response of Nile tilapia (*Oreochromis niloticus*) exposed to heat stress	*Oreochromis niloticus*	Heat stress	Cortisol - glucose - SOD - catalase - GPx - MDA	Acute	Intestine	https://doi.org/10.1016/j.jtherbio.2019.102500
Differential regulation of endoplasmic reticulum stress-induced autophagy and apoptosis in two strains of gibel carp (*Carassius gibelio*) exposed to acute waterborne cadmium	*Carassius gibelio*	Exposed to acute waterborne cadmium	Cortisol - glucose - mRNA of metallothionein and antioxidant signaling - mRNA and bip and eif2a - ire1 - perk - xbp1 - atf4 - atg12 - beclin1 - lc3b - bcl2 -	Chronic	Blood samples and liver	https://doi.org/10.1016/j.aquatox.2020.105721
Divergent gene expression in the gills of juvenile turbot	*Psetta maxima*	hypercapnia	IL-1B - LMP7 - Grim9 - DFAD - ApoE - COX - EF1a - STAT2	Chronic	Gill	https://doi.org/10.1016/j.aquatox.2018.10.023

(*Psetta maxima*) exposed to chronic severe hypercapnia indicates dose-dependent increase in intracellular oxidative stress and hypoxia						
Early stress exposure on zebrafish development: effects on survival, malformations and molecular alterations	*Danio rerio*	Temperature (heat and cold) Chemicals - mechanical - osmotic - pH	micro-RNA 29a - rarga - rxrba - rxrbb - col2a1a - col2a1b - col6a2 - col6a3 - col11a1a	Acute		https:// doi. org/ 10. 1007/ s10695-024- 01355-0
Effect of amino acid supplementation and stress on expression of molecular markers in meagre (*Argyrosomus regius*)	*Argyrosomus regius*	confinament and netting (being chased with a net)	crh - crh-bp - trh - sl - prl - gh - pomc-a - Hsp70	Acute	Blood samples and brain, hypophysis and liver tissues	https://doi.org/10.1016/j.aquaculture.2020.736238
Effect of excessive doses of oxytetracycline on stress-related biomarker expression in coho salmon	*Oncorhynchus kisutch*	excessive doses of oxytetracycline	Hsp70 - glutathion	Acute	Plasma and liver	10.1007/s11356-015-4898-4
Effect of Fish Stock Density on Hormone Genes Expression from Brain and Gastrointestinal Tract of *Salmo salar*	*Salmo salar*	Stock Density	NPY - CGRP - VIP - Prk1 - Cortisol - glucose	Chronic	Brain and gastrointestinal tract and liver	https://doi.org/10.3390/ani12091174

Effects of cold stress on juvenile *Piaractus mesopotamicus* and the mitigation by β-carotene	*Piaractus mesopotamicus*	cold temperatures	Hematological parameters- protein - colesterol - triglycerides - glucose - antioxydant enzymes	Acute	Liver - intestine - gills - brain - muscle	https://doi.org/10.1016/j.jtherbio.2019.102497
Effects of heat stress on histopathology, antioxidant enzymes, and transcriptomic profiles in gills of pikeperch *Sander lucioperca*	*Sander lucioperca*	heat stress	Superoxide dismutase (T-SOD) - glutathione peroxidase (GPx) - catalase (CAT) - glutathione reductase (GR) - the malondialdehyde (MDA) - Cu–Zn SOD, Mn SOD and CAT,	Acute	Gills	https://doi.org/10.1016/j.aquaculture.2020.736277
Effects of hypoxia stress on the intestinal microflora of juvenile of cobia (*Rachycentron canadum*)	*Rachycentron canadum*	hypoxia	Pseudoalteromonas and Actinomyces	Chornic	Microflora study	https://doi.org/10.1016/j.aquaculture.2021.736419
Effects of Low Stocking Densities on Zootechnical Parameters and Physiological Responses of Rainbow Trout (*Oncorhynchus mykiss*) Juveniles	*Oncorhynchus mykiss*	Low Stocking Densities	il-1β (1,2,3), il-4, il-6, il-8, il-10 and il-11 - tnf-αa - (crf) - hormone receptor (crhr1, crhr2), melanocortin 2 receptor (mc2r), lysozyme 2 (lyzII), inter-<br>feron gamma (ifn-γ1), heat shock proteins (hsp30, hsp70), interferon regulatory factor (irf-1airf-1b, irf3, irf4, irf-5a, irf-5b and irf9) _ Cortisol - lactoto - plasma lysozyme	Chronic	Blood cells	10.3390/biology10101040

Environmental salinity differentiates responses to acute hypothermal stress in milkfish	*Chanos chanos*	hypothermal stress	leptin A - sod1 - catalase	Acute	Liver - blood	https://doi.org/10.1016/j.scitotenv.2024.176643
Evaluation of physiological stress of grass carp chronically exposed to enrofloxacin based on IBR Index	*Ctenopharyngodon idella*	Exposed to Enrofloxacin	stress biomarkers (including superoxide dismutase (SOD), catalase (CAT), reduced glutathione (GSH), and malondialdehyde (MDA)), neurotoxicity indicators (including acetyl-cholinesterase (ACHE) activity, nitric oxide (NO)), and digestive enzyme activities (including lipase(LPS), amylase (AMS) enzymes). sod1 - LPL - ATGL - AMY	chronic	Integred biomarker reponse	10.1007/s11270-023-06574-4
Exploring the effects of rearing densities on epigenetic modifications in the Zebrafish gonads	*Danio rerio*	Rearing densities	dnmt1, cyp19a1a, dmrt1, cyp11c1, hsd17b1, and hsd11b2,	Acute	Gonads	10.3390/ijms242116002
Expression of Hsp70, Igf1, and three oxidative stress biomarkers in response to handling and salt treatment at different water temperatures in Yellow Perch, *Perca flavescens*	*Perca flavescens*	Handling stress - salt treatment - temperature	heat shock protein (hsp70), insulin-like growth factor 1 (Igf1), glutathione peroxidase (Gpx), superoxide dismutase 1 (Sod1), andglutathione reductase (Gsr)	Acute	blood and liver samples	10.3389/fphys.2017.00683

Functional feeds to tackle Meagre (*Argyrosomus regius*) stress: Physiological responses under acute stressful handling conditions	*Argyrosomus regius*	Handling conditions	TNF-α and IL-1β - IL-10 - HSP70 and HSP90 - GPX, GR, and G6PDH. Hepatic SOD, GR, and G6PDH activities	Acute	hematologic parameters - muscle	10.3390/md19110598
Genetic pathways underpinning hormonal stress responses in fish exposed to short- and long-term warm ocean temperatures	*Dicentrarchus labrax*	Temperature	Cortisol - (nr3c1, nr3c2 and hsd11b2) - crhr1, mc2r, mc5r, trh or trhr,	Chronic	Hormone quantification	https://doi.org/10.1016/j.ecolind.2020.106937
Genome-wide association analysis of heat tolerance in the northern pike (*Esox Lucius*)	*Esox Lucius*	Temperature	*atp13a3 - clstn2*	Acute	GWAS	https://doi.org/10.1016/j.aquaculture.2022.738459
Growth performance, physiological responses to hypoxia and flesh quality of Chilean croaker (*Cilus gilberti*) stocked at different densities	*Cilus gilberti*	Hypoxia and stock densities	HSP70 - IGF-1 - glucose - lactate - TAG	Acute		https://doi.org/10.1016/j.aquaculture.2020.735316
Health and welfare of Atlantic Salmon in FishGLOBE V5 – a Novel Closed Containment System at Sea	*Salmo salar*	FishGLOBE V5 – a Novel Closed Containment System at Sea	cortisol - glucose - magnesium - keratin, type I cytoskeletal 20 - epithelial membrane protein 3 - hrombospondin-2 - cadhl, emp3, and thbs2 - heat	Chronic	Plasma - gills - skin	https://doi.org/10.3389/fanim.2022.871433

			shock protein 90 - mucin 5b - mucin 2			
High throughput sequencing of MicroRNA in rainbow trout plasma, mucus, and surrounding water following acute stress	*Oncorhynchus mykiss*	3 min exposition to air	Cortisol - glucose - lactate - ssa-miR-30b-5p - miR-30b - miR-26a - miR-16b-5p and miR-16c-5p	Acute	Plasma and epidermal mucus	https://doi.org/10.3389/fphys.2020.588313
High-Temperature stress effect on the Red Cusk-Eel (*Geypterus chilensis*) Liver: Transcriptional modulation and oxidative stress damage	*Geypterus chilensis*	High-Temperatures	HSP 90 - HSP 40 - HSP 70 - HSP 60 - Leptin - casp3 - ddit4 - gpx7 - msh2 - msh3 - clql1 - ccl290 - atg12 - atg4b - atg4b -c3	Chronic	Liver	10.3390/biology11070990
HSP60 and HSP90β from blunt snout bream, *Megalobrama amblycephala*: Molecular cloning, characterization, and comparative response to intermittent thermal stress and Aeromonas hydrophila infection	*Megalobrama amblycephala*	Termal stress and infection	HSP60 and HSP90β	Acute	Liver	https://doi.org/10.1016/j.fsi.2017.12.046
Hypothermal stress induced differential expression profiles of the immune response	*Chanos chanos*	Hypothermal stress	Ccwap65-1 and Ccwap65-2	Acute	Liver	https://doi.org/10.1016/j.fsi.2017.09.012

gene, warm-temperature-acclimation associated 65-kDa protein (Wap65), in the liver of fresh water and seawater milkfish, *Chanos chanos*						
Identification of differentially expressed proteins of brain tissue in response to methamidophos in flounder (*Paralichthys olivaceus*)	*Paralichthys olivaceus*	Methamidophos exposition	HSP 90 - Gzmk - GR - Cyt c -Ape	Acute		https://doi.org/10.1016/j.fsi.2015.03.028
Identification, classification and functional characterization of HSP70s in rainbow trout (*Oncorhynchus mykiss*) through multi-omics approaches	*Oncorhynchus mykiss*	Acute thermal stress and Ichthyophthirius multifiliis infection - (hypoxia, thermal, and overcrowding stress)	HSP70	Acute		https://doi.org/10.1016/j.fsi.2021.12.059
Impact of different levels of handling on *Solea senegalensis* culture: effects on growth and molecular markers of stress	*Solea senegalensis*	Handling	Cortisol - hsp90aa - grl - gr2 - casp6	Acute	Blood plasma	https://doi.org/10.1007/s10695-023-01239-9

Long term salinity disrupts the hepatic function, intenstinal health, and gills atioxidative status in *Nile Tilapia* stressed with hipoxia	*Oreochromis niloticus*	Hypoxia-salinity	Il8 - Il-1B - Ifn-y - Tnf-a - Caspase 3 - Il-10	Chornic	Liver - intestine - gills-	https://doi.org/10.1016/j.ecoenv.2021.112412
Long term stress induced cortisol dowrugulation, growth reduction and cardiac remodeling in Atlantic Salmon (*Salmo salar*)	*Salmo salar*	Stressors - chasing - crowding - netting - air exposure - temperature shock	Cortisol - crh - pomca1 - pomca2 - gr - star - mr - 11B-hasd2 -	Chornic	Blood - hypothalamus - pituitary - kidney	10.1242/jeb.246504
Lowering temperature to 1 C results in physilogical changes, stress and mortality in cultured Atlantic Salmon (*Salmor salar*)	Salmo salar	Lowerin temperature	Hsp70 - Hsp90 - cortisol - serpinh1 - cribp	Chronic	Liver - blood -	https://doi.org/10.1016/j.aquaculture.2023.739313
Molecular cloning of myosin chaperone uncoordinate-45B (UNC-45B) and its heat stress response in the muscle of Pacific bluefin tuna *Thunnus orientalis*	*Thunnus orientalis*	Heat stress	UNC-45B	Acute		https://doi.org/10.1007/s12562-021-01557-y
Muscle cortisol levels, expression of Glucocorticoid Receptor and Oxidative Stress	*Argyrosomus regius*	Transport stress	Crortisol - gr - HNE - HSP70 - NT - 8-OHdG	Acute	Muscle - liver	10.3390/ani11041160

Markers in the teleost fish *Argyrosomus regius* exposed to transport stress						
Muscle transcriptome sequencing revealed thermal stress–responsive regulatory genes in farmed Rohu, *Labeo rohita* (Hamilton, 1822)	*Labeo rohita*	Heat stress	SERPINE1(HSP47) - HSP70 - HSP90alpha - Rano class II histocompatibility A beta, PGC-1 and ERR-induced regulator - proto-oncogene c-Fos - myozenin2 - alpha-crystallin B chain-like protein - angiopoietin like protein 8, and acetyl-CoA carboxylases	Chronic	Muscle tissue	10.1007/s10126-023-10259-8
Physiological responses to cold and starvation stresses in the liver of yellow drum (*Nibea albiflora*) revealed by LC-MS metabolomics	*Nibea albiflora*	Cold and starvation stresses	glutamate and GSSG	Acute	Liver	10.1016/j.scitotenv.2020.136940
Potential role of dietary zinc on gene regulation of growth performance and immunity in *Pangasianodon hypophthalmus* against multiple stresses	*Pangasianodon hypophthalmus*	Exposition to arsenic - ammonia - pollution - high temperature	Cortisol, inducible nitric oxide synthase (iNOS), heat shock protein (HSP70), and metallothionine (MT), catalase (CAT), superoxide dismutase (SOD), and glutathione peroxidase (GPx) - caspase 3a (CAS 3a), tumour necrosis factor (TNFα), interleukin (IL),	Chronic	Giver and gill	https://doi.org/10.1016/j.aquaculture.2023.740271

			and toll-like receptors (TLR)			
Repeated hormonal induction of spermiation affects the stress but not the immune response in pikeperch (*Sander lucioperca*)	Sander lucioperca	Hormonal injection	cortisol - glucose - il1 - c3 - hamp - tnf-a - lys	Acute	Blood- kidney	https://doi.org/10.1016/j.fsi.2020.03.057
Dual effects of dietary *Lactobacillus helveticus* and chlorogenic acid on growth performance, digestibility, immune-antioxidant capacity and resistance against heat stress of juvenile common carp	*Cyprinus carpio*	Heat stress	CGA - Lh - ACH50 - MDA - AST - ALT - ALP - Glucose - Cortisol	Chronic	Blood - liver	
Salinity Tolerance in Freshwater Drum (*Aplodinotus grunniens*): Investigating Biochemical, Antioxidant, Digestive Enzyme, and Gene Expression Responses to Acute Salinity Stress	*Aplodinotus grunniens*	Salinity stress	Aspartate aminotransferase - alanine aminotransferase - malondialdehyde - HSP70 - HSP90 -	Acute	Gills and liver	10.3390/ani15071015
Scales tell a story on the stress history of fish	*Cyprinus carpio L.*	Netting - air exposure - sudden temperature	Cortisol	Chronic	Blood and scales	10.1371/journal.pone.0123411

		drop - chasing - confinement				
Screening and identification of the biomarkers applied for the evaluation of acute and chronic thermal tolerance ability in largemouth bass (*Micropterus salmoides*)	*Micropterus salmoides*	Cold and heat stress	cortisol, blood glucose, and lactate levels; activities of superoxide dismutase (SOD), glutathione peroxidase (GPX), catalase (CAT), glucose kinase (GK), pyruvate kinase (PK), lactate dehydrogenase (LDH), and glucose 6 phosphatase (G6Pase); and the expressions of hsp70 and hsp90 in both NLMB and FLMB	Chronic and acute	Blood samples	https://doi.org/10.3390/ani14101435
Silybin prevented avermectin-induced cardiotoxicity in carp by modulating oxidative stress, inflammation, endoplasmic reticulum stress, mitochondrial apoptosis, and autophagy	*Cyprinus carpio*	Toxicity	NF-κB - Nrf2 - Keap1 - CK - CK-MB -	chronic	Blood samples	https://doi.org/10.1016/j.fsi.2024.109624
Stocking density affects growth, feed utilisation, metabolism, welfare and associated mRNA transcripts in liver and muscle of rainbow trout more	*Oncorhynchus mykiss*	Stocking density	antioxidative enzymes (sod, cat, gst, g6pd) and stress-related molecular chaperone hsp90 - (ghr, igf1 igf2) - (ampk1, hsp70, mhc1 and tnfa)	Chronic	Blood samples	https://doi.org/10.1016/j.aquaculture.2024.741717

pronouncedly than dietary fish meal inclusion level						
Stocking density affects immune and stress-related gene expression of Butter catfish (*Ompok bimaculatus*) fry in biofloc landscapes	*Ompok bimaculatus*	Stocking density	catalase, glutathione-S-transferase, and malondialdehyde - HSP70, IL-1, and IL-20 - Nrf-2 and Tlr-9 - glucose and cortisol	chronic	Blood and gut liver and muscle	https://doi.org/10.1016/j.fsirep.2023.100112
Stress and gut responses of post-smolt Atlantic salmon (*Salmo salar*) to elevated levels of hydrogen sulphide	*Salmo salar*	Exposure to H2S	sulfide:quinone oxidoreductase 2 (sqor2) - bcl-associated x protein (bax) - caspase-3a (casp3a) - cluster of differentiation 4 (cd4) - cortisol	Chronic	Blood and distal gut	https://doi.org/10.1016/j.aquaculture.2023.740467
Sulforaphane modulates some stress parameters in TPT-exposed *Cyprinus carpio* in relation to liver metabolome	Cyprinus carpio	Exposed to triphenyltin	HSP70 - Hsp90 - SOD - CAT - GPX	Chronic	Blood and liver samples	10.1016/j.ecoenv.2024.116882
The Atlantic salmon's stress- and immune-related transcriptional responses to moderate hypoxia, an incremental temperature increase, and these challenges combined	*Salmo salar*	Moderate hypoxia- incremental temperature - combined	hsp47 - hsp90aa1 - hsp70 - jak2 - jund - hcn1 - txn - pdk3- cirbp - prdx6 - ucp2 - rrarga - cyp1a1 - hif1a - calm - dnmt1	Chronic		10.1093/g3journal/jkab102

The potential for heat shock exposure during early development to alter the molecular responses to subsequent exposure to heat stress in the rainbow trout	*Oncorhynchus mykiss*	Heat shock exposure	hsp70a - hsp70b	Acute		https://doi.org/10.1016/j.cbpa.2025.111817
The transcriptomic responses of Atlantic salmon (*Salmo salar*) to high temperature stress alone, and in combination with moderate hypoxia	*Salmo salar*	High temperature and moderate hypoxia	hsp90aa1, serpinh1 - casp8, jund, jak2 - apod, c1ql2, epx - cirbp, cyp1a1, egln2, gstt1, hif1α, prdx6, rraga, ucp2	Chronic	Liver - blood	https://doi.org/10.1594/PANGAEA.913696)
Thermal stress of ambient temperature modulate expression of stress and immune-related genes and dna fragmentation in nile tilapia (*Oreochromis niloticus* (linnaeus, 1758))	*Oreochromis niloticus*	Thermal stress	hsp70 - hsp27 - MT - GST - IL1 - IL8 - TLR7 -		Gills - liver - blood	http://dx.doi.org/10.15666/aeer/1503_13431354
Tissue origin of circulating microRNAs and their response to nutritional and environmental stress in rainbow trout (*Oncorhynchus mykiss*)	*Oncorhynchus mykiss*	Hypoxia and nutritional stress	miR-122-5p, miR-184-3p, miR-192-5p and miR-194a-5p - miR-210-3p, miR-153a-3p and miR-218c-5p	Acute	Blood and epidermal mucus	https://doi.org/10.1016/j.scitotenv.2022.158584

Toward new biomarkers of cold tolerance: microRNAs regulating cold adaptation in fish are differentially expressed in cold-tolerant and cold-sensitive Nile tilapia (*Oreochromis niloticus*)	*Oreochromis niloticus*	Cold stress	miR-9-3p, miR-135c, miR-9-5p, miR-30b, miR-122, miR-92a, IGF-a, SCD, cortisol		Blood - brain - liver samples	https://doi.org/10.1016/j.aquaculture.2024.740942
Toxicological effects of cadmium on the immune response and biomineralization of larval flounder Paralichthys olivaceus under seawater acidification	*Paralichthys olivaceus*	Exposed to cadmium	hsp70 - mt - lzm	Chronic	Larvae	https://doi.org/10.1016/j.chemosphere.2021.132919
Transport stress changes blood biochemistry, antioxidant defense system, and hepatic HSPs mRNA expressions of Channel catfish, *Ictalurus punctatus*	*Ictalurus punctatus*	Transport stress	Cortisol, glucose, cholesterol, triglyceride, aspartate aminotransferase, glutathione peroxidase and catalase, HSP70, HSP90.	Acute	Blood samples	https://doi.org/10.3389/fphys.2018.01628
Unraveling vasotocinergic, isotocinergic and stress pathways after	Sparus aurata	food deprivation and stocking density	crh - crhbp - avtrv1a - avtrv2 - itr - cortisol	Chronic	Blood, liver, hypotalamus, pituitary and head-kidney	https://doi.org/10.1016/j.cbpa.2017.10.012

food deprivation and high stocking density in the gilthead sea bream						
What metabolic, osmotic and molecular stress responses tell us about extreme ambient heatwave impacts in fish at low salinities: The case of European seabass, *Dicentrarchus labrax*	*Dicentrarchus labrax*	Different salinities and heat wave	glucose - lactate - triglycerides - GPT - GOT - Cortisol - BUN - HSP70 - Igf1	Chronic	Blood	https://doi.org/10.1016/j.scitotenv.2020.141458
